# Perianal fistulizing Crohn’s disease increases infliximab clearance and causes low infliximab exposure (ATLANTIC)

**DOI:** 10.1093/ecco-jcc/jjag151

**Published:** 2026-10-23

**Authors:** Lieven G M Mulders, Suzanne I Anjie, Manon Chevallot-Beroux, Floris Loeff, Manon E Wildenberg, Ron A A Mathôt, Geert R A M D’Haens, Krisztina B Gecse

**Affiliations:** Department of Gastroenterology and Hepatology, Amsterdam UMC, University of Amsterdam, Amsterdam Gastroenterology Endocrinology Metabolism, Amsterdam, The Netherlands; Department of Gastroenterology and Hepatology, Amsterdam UMC, University of Amsterdam, Amsterdam Gastroenterology Endocrinology Metabolism, Amsterdam, The Netherlands; Biologics Laboratory, Sanquin Diagnostic Services, Amsterdam, The Netherlands; Biologics Laboratory, Sanquin Diagnostic Services, Amsterdam, The Netherlands; Tytgat Institute for Liver and Intestinal Research, Amsterdam UMC, University of Amsterdam, Amsterdam Gastroenterology Endocrinology Metabolism, Amsterdam, The Netherlands; Department of Hospital Pharmacy and Clinical Pharmacology, Amsterdam UMC, University of Amsterdam, Amsterdam, The Netherlands; Department of Gastroenterology and Hepatology, Amsterdam UMC, University of Amsterdam, Amsterdam Gastroenterology Endocrinology Metabolism, Amsterdam, The Netherlands; Department of Gastroenterology and Hepatology, Amsterdam UMC, University of Amsterdam, Amsterdam Gastroenterology Endocrinology Metabolism, Amsterdam, The Netherlands

**Keywords:** perianal fistulizing Crohn’s disease, infliximab, pharmacokinetics, therapeutic drug monitoring

## Abstract

**Background and aims:**

In perianal fistulizing Crohn’s disease (PFCD), higher infliximab (IFX) serum concentrations are associated with improved outcomes. PFCD patients frequently require dose intensification to reach recommended therapeutic targets. IFX pharmacokinetics (PK) in PFCD have not been previously characterized. We hypothesized that drug clearance (CL) is increased in PFCD compared to luminal Crohn’s disease (LCD).

**Methods:**

Patients with PFCD and LCD starting standard-dose intravenous IFX (5 mg/kg at weeks 0, 2, and 6, thereafter every 8 weeks) were enrolled. At every visit, serum samples were collected until week 52 to measure IFX trough levels (TL) and anti-drug antibodies. A population PK model was constructed using nonlinear mixed-effects modeling and adjusted for clinically relevant covariates.

**Results:**

A total of 148 patients were included (62 PFCD, 86 LCD) with 756 serum samples analyzed. IFX PK was described by a two-compartment model with linear elimination. After adjusting for dose, weight, serum albumin, and anti-drug antibodies, population PK modeling revealed a 23% increase in IFX CL among patients with progressive perianal fistulas during the first 14 weeks (*P* = .007). During induction, recommended target IFX TL was achieved in fewer PFCD than LCD patients (43% vs 57%, *P* = .04). At week 14, PFCD patients had lower median IFX TL than LCD (2.9 vs 4.8 μg/mL, *P* = .02), with fewer PFCD patients reaching phenotype-specific target TL (9% vs 46%, *P* < .001) compared to LCD.

**Conclusion:**

The presence of perianal fistulas increases IFX CL in patients with Crohn’s disease, resulting in significantly lower and frequently subtherapeutic serum concentrations after standard induction dosing.

## Graphical abstract

**Graphical Abstract:**
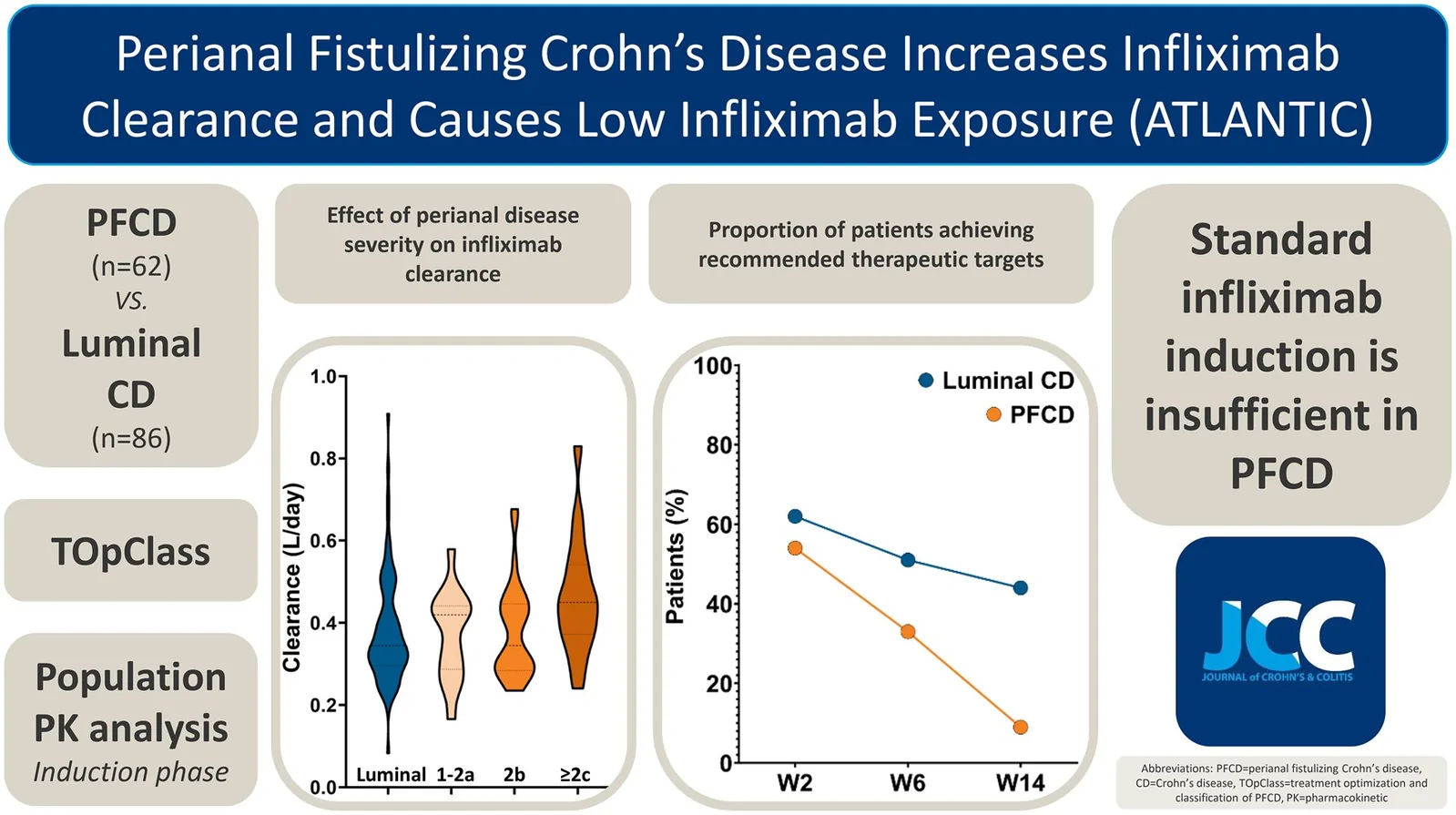

## 1. Introduction

Perianal fistulizing Crohn’s disease (PFCD) is a debilitating phenotype known for relapsing behavior, poor long-term prognosis, and negative impact on quality of life.^1^^,^^2^ The incidence of PFCD is as high as 37% in patients with Crohn’s disease (CD) at tertiary care centers, and up to 90% in patients with rectal inflammation.^3^^,^^4^ Patients with PFCD typically need more frequent hospital visits and invasive procedures, and treatment is associated with higher healthcare costs, underpinning the clinical and economic burden of this disease.^5^^,^^6^ Despite advances in therapeutic approaches, management remains challenging and continues to rank highly among top research priorities in inflammatory bowel disease (IBD).^7^

Anti-tumor necrosis factor (TNF) therapies (ie, infliximab, adalimumab) have been the cornerstone of treatment for moderate-to-severe CD, including PFCD, for more than two decades.^8–11^ Nevertheless, primary treatment failure is reported in up to 66% of patients, highlighting the need for improved therapeutic strategies.^12^^,^^13^ Infliximab was the first monoclonal antibody to be approved for the treatment of CD and PFCD for both induction and maintenance treatment.^8^^,^^9^^,^^14–16^

Intravenous infliximab pharmacokinetics (PK) are characterized by rapid intravenous distribution and limited extravascular distribution, and the clearance is primarily influenced by inflammation-driven catabolism.^17–19^ Increased clearance correlates with elevated serum C-reactive protein (CRP), decreased serum albumin, and anti-drug antibody (ADA) formation, leading to substantial inter- and intra-individual variability in serum drug concentrations.^20–24^ Such variability complicates standardized dosing regimens, emphasizing the clinical value of therapeutic drug monitoring (TDM) to enhance treatment efficacy and patient outcomes.

Several studies have demonstrated a positive pharmacodynamic association between infliximab drug exposure and clinical outcomes, including clinical and radiological therapy response.^25–28^ Although most data derive from retrospective cohorts during maintenance therapy with infliximab or adalimumab, a post-hoc analysis of the pivotal ACCENT-II trial in PFCD patients suggested that specific induction-phase infliximab trough level (TL) thresholds (≥20.2 µg/mL at week 2, ≥15 µg/mL at week 6, and ≥7.2 µg/mL at week 14) were associated with composite remission at week 14.^29^ Target TLs of ≥10 µg/mL during maintenance are frequently recommended in clinical practice and form the basis of TDM-based treatment strategies.^30^ Nonetheless, most patients with moderate-to-­severe CD, including PFCD, require early intensification or accelerated infliximab regimens to reach these therapeutic thresholds.^31^^,^^32^ However, TDM is typically initiated at week 14 or later, meaning that dose adjustments are often reactive rather than proactive, potentially missing a critical window for early treatment optimization. Delayed attainment of therapeutic infliximab levels during the induction phase may contribute to slower therapeutic responses and poorer outcomes.

We therefore hypothesized that standard infliximab induction dosing is insufficient in PFCD. This study aimed to investigate infliximab PK differences during the induction phase (weeks 0-14) among patients with PFCD and luminal disease only.

## 2. Methods

### 2.1. Design and participants

ATLANTIC was an investigator-initiated, prospective, single-center, cohort study performed at Amsterdam UMC, The Netherlands. Amsterdam UMC is a tertiary referral center and has a dedicated PFCD clinic.

The study enrolled patients with diagnosed CD aged 16 years and older starting on intravenous infliximab between December 2015 and January 2024. Patients were stratified into groups with (1) perianal fistulizing disease with or without luminal activity (PFCD), or (2) luminal Crohn’s disease only (LCD). Patients were participants in the Amsterdam UMC IBD Biobank (FUTURE IBD). Consecutive patients with a confirmed diagnosis of CD were screened for inclusion at the time of initiating treatment with advanced medical therapies and provided signed informed consent.

This study was performed according to the principles of the Declaration of Helsinki (version 2013), Good Clinical Practice guidelines, and received IRB approval from the Medical Ethical Committee at the Amsterdam UMC (METC Amsterdam UMC 2014.327 and 2024.0462). The study is registered in the International Clinical Trials Registry Platform, NL-OMON50413.

### 2.2. Study procedures

The study protocol was based on standard intravenous infliximab induction regimen at a dose of 5 mg/kg at weeks 0, 2, and 6, followed by maintenance infusions at an interval of 8 weeks. Intensified dosing was allowed at the physician’s discretion, based either on clinical indication (reactive TDM) or on pre-emptive serum concentrations (proactive TDM). Concomitant treatment with any other medication, including thiopurine, mercaptopurine, thioguanine, methotrexate, budesonide, hydrocortisone, and prednisone, was allowed.

At each infusion visit, pre-infusion serum samples, blood tests, and fecal samples were collected to assess inflammatory markers, including CRP, serum albumin, white blood cell count, and fecal calprotectin. Concomitant medication use was recorded at each visit. Serum sampling continued throughout the 52-week follow-up period. All serum samples were stored at −70 °C and centrally analyzed for infliximab TLs by Sanquin Diagnostic Services (Amsterdam, The Netherlands) using a previously validated enzyme-linked immunosorbent assay (ELISA).^33^ The lower limit of quantification (LLOQ) was 0.03 µg/mL. ADAs against infliximab were measured if infliximab TLs were below 1.0 µg/mL, using a drug-sensitive radioimmunoassay antigen-binding test.^33^ The lower limit of detection was 12 arbitrary units (AU)/mL, the LLOQ was 30 AU/mL, and the upper limit of quantification was 875 AU/mL. Data were collected in an electronic database, Castor EDC (Amsterdam, The Netherlands).

### 2.3. Outcomes

The primary outcome was infliximab clearance during the induction phase (weeks 0-14), estimated using nonlinear mixed-effects modeling (NONMEM) and compared between patients with PFCD and LCD. Secondary outcomes included differences in infliximab TLs, the proportion of patients achieving predefined target TLs at weeks 2, 6, and 14, the incidence of ADAs, and differences in additional PK parameters during the induction phase. Fistula clinical status (active/response/remission/) was compared with infliximab exposure at week 14 as an exploratory post-hoc analysis (Supplementary Results). Infliximab target TLs were defined as 20 μg/mL at week 2, 15 μg/mL at week 6, and 10 μg/mL at week 14 for PFCD patients, and 5 μg/mL at week 14 for LCD patients.^25–29^ These pragmatic targets, derived from a post-hoc trial analysis and observational literature, are used as clinical thresholds rather than validated outcome-discriminating cutoffs.

### 2.4. Population PK model development

All available infliximab concentration data collected, including trough and intermediate samples, were included in the population PK model to optimize parameter estimations. Infliximab concentration–time profiles were described using a two-compartment population PK model in terms of clearance, intercompartmental clearance (*Q*), and central and peripheral volumes of distribution (*V*_c_ and *V*_p_). Because not all structural parameters could be reliably estimated from our sampling design, *Q* and *V*_p_ were fixed to values previously reported by Hanzel et al. in a population PK model of infliximab developed in adults with IBD receiving intravenous induction and subcutaneous maintenance therapy.^24^ The M3 method was applied to incorporate samples with concentrations under the LLOQ.^34^ Inter-patient variability of clearance was modeled assuming a log-normal distribution of the individual values. The residual error was modeled with a combination of an additive and proportional error.

After development of the structural population PK model, associations between patient characteristics and clearance were evaluated using only the induction phase samples (weeks 0-14). Based on published literature, clinical interest, and mechanistic plausibility, covariates for testing were predefined (Table S1). Perianal fistulizing disease was classified by the TOpClass classification as simple or suitable for repair (category 1 or 2a), symptom control (2b), and progressive disease (≥2c-i).^35^^,^^36^

Correlations between covariates were assessed; variables with absolute correlation coefficient values greater than 0.30 were not simultaneously included as predictors in the model. The association of continuous covariates and PK parameters was described using power models. Categorical covariates entered the model as power functions with a separate dichotomous (0, 1) covariate serving as an on–off switch for the effect.

The fit of the model to data was evaluated on the basis of several criteria. Relative standard errors of the estimated parameters and standard goodness-of-fit plots were inspected. For the covariate analysis, the log-likelihood ratio was applied with a drop of 6.7 points in objective function value (OFV) corresponding to a *P* < .01 (1 df). The final model was evaluated using a predicted-corrected visual predictive check. Bootstrapping was performed to obtain nonparametric estimates of uncertainty for the final model (*n* = 1000 bootstraps).

### 2.5. Statistical analyses

No formal power calculation was performed, as traditional power calculations are not directly applicable to population PK analyses. Instead, a sample size of approximately 50 patients per group was considered adequate to estimate infliximab clearance and assess covariate effects with acceptable precision, based on prior population PK studies in IBD and established simulation-based recommendations.^37^ This sample size supports robust estimation of interindividual variability and model stability in NONMEM.

Analyses were conducted according to the intention-to-treat principle, meaning that all patients receiving at least one infliximab infusion were included. Descriptive statistics were provided with means and standard deviation (SD), or medians and interquartile range (IQR) for continuous variables, and number and frequencies (%) for categorical variables. Inferential statistics included comparisons of observed infliximab TLs at weeks 2, 6, and 14 and ADA incidence between PFCD and LCD cohorts, using the two-sided Mann–Whitney U or unpaired t-test depending on data distribution.

Binary outcomes (eg, achieving a predefined TL) were first compared using Fisher’s exact test. Univariable and multivariable logistic regression were used to estimate odds ratios (OR) and 95% confidence intervals (CI). To facilitate interpretation and given the repeated-measures design, robust Poisson regression was performed as the primary modeling strategy to directly estimate population-averaged risk ratios (RR) and adjusted risk ratios (aRR). A *P*-value of <.05 was considered statistically significant. Statistical analyses were performed using R (version 2024.12.0 + 467) and relevant packages and visualized using GraphPad Prism (version 10). Population PK modeling was performed using NONMEM software (NONMEM, version 7.5, ICON PLC, Ireland).

## 3. Results

### 3.1. Patient and disease characteristics

A total of 151 patients were enrolled, and 148 were included in the intention-to-treat analysis (Figure S1). The study population consisted of 62 patients with PFCD and 86 patients with only luminal CD. The baseline characteristics of the patients with PFCD were similar to those of patients with LCD, except for luminal disease characteristics (Table 1). LCD patients had higher fecal calprotectin and Simplified Endoscopic Activity Score for Crohn’s Disease (SES-CD) at baseline, and more often used concomitant immunomodulators. Induction dosing was similar, with only 2% of PFCD and 1% of LCD patients receiving intensified induction (10 mg/kg), and 21% of PFCD and 15% of LCD patients receiving an additional infusion at week 10. Intensified maintenance regimens (defined as intervals ≤6 weeks and/or doses ≥7.5 mg/kg) were initiated in 30% of PFCD and 20% of LCD patients.

**Table 1.** Baseline demographics and disease characteristics.

	**PFCD (*N*** = **62)**	**LCD (*N*** = **86)**
**Demographics**		
** Age (years), median [IQR]**	31 [22-46]	34 [25-49]
** Female, *n* (%)**	31 (50)	54 (63)
** Body weight (kg), median [IQR]**	69 [60-78]	72 [63-81]
** BMI (kg/m^2^), median [IQR]**	22.1 [19.7-24.9]	23.7 [21.6-25.8]
**Disease characteristics**		
**Age at diagnosis, *n* (%)**		
** <17 y/o**	14 (23)	19 (22)
** 17-39 y/o**	42 (68)	53 (62)
** >40 y/o**	6 (10)	15 (17)
**Luminal disease location, *n* (%)**		
** Ileal**	9 (15)	19 (22)
** Colonic**	13 (21)	31 (36)
** Ileocolonic**	36 (58)	36 (42)
** No luminal disease**	4 (6)	0
**Luminal disease behavior, *n* (%)**		
** Inflammatory**	39 (65)	51 (59)
** Stenotic**	11 (18)	23 (27)
** Penetrating**	10 (17)	12 (14)
**Upper GI involvement, *n* (%)**	13 (21)	14 (17)
**Disease duration (years), median [IQR]**	8 [2-15]	8 [3-15]
**Previous biological treatment, *n* (%)**	32 (52)	46 (53)
**Previous infliximab treatment, *n* (%)**	19 (31)	14 (16)
**Proctitis, *n* (%)**	41 (66)	48 (56)
**Previous bowel resection, *n* (%)**	19 (31)	25 (29)
**Smoking history, *n* (%)**		
** Former**	6 (10)	13 (15)
** Active**	10 (16)	24 (28)
**Disease activity at baseline**		
** SES-CD at baseline, median [IQR]**	8 [3-15]	10 [7-16]
** CRP at baseline (mg/L), median [IQR]**	5.2 [2.2-20.0]	5.4 [1.5-17.5]
** Fecal calprotectin at baseline, µg/g, median [IQR]**	869 [176-1851]	1216 [308-2982]
** Serum albumin at baseline, g/L, median [IQR]**	41 [38-44]	41 [38-44]
**TOpClass classification, *n* (%)**		
** Minimal disease or suitable for repair (TOpClass 1 or 2a)**	15 (25)	n/a
** Symptom control (TOpClass 2b)**	30 (48)	n/a
** Severe, progressive (TOpClass 2c-i, 2c-ii, 4)**	17 (27)	n/a
**Treatment characteristics**		
** Concomitant immunomodulator or methotrexate, *n* (%)**	41 (66)	71 (83)
** Concomitant corticosteroids, *n* (%)**	23 (37)	38 (44)
** Infliximab starting dose, mg/kg, mean (95% CI)**	5.2 (5.0-5.4)	5.1 (5.0-5.2)

Abbreviations: PFCD, perianal fistulizing Crohn’s disease; LCD, luminal Crohn’s disease; IQR, interquartile range; BMI, body mass index; GI, gastrointestinal; SES-CD, Simplified Endoscopic Activity Score for Crohn’s Disease; CRP, C-reactive protein.

### 3.2. Population pharmacokinetics and infliximab clearance

A total of 756 serum samples were collected across weeks 2-52, including 387 pre-infusion samples during induction (week 2: *n* = 121; week 6: *n* = 129; and week 14: *n* = 137). Sample contribution was proportional across PFCD (*n* = 325) and LCD (*n* = 431) groups, with no significant differences in missing data rates at key induction timepoints (Table S2).

For the population PK analysis, data were available from the intention-to-treat dataset (148 patients). A total of 398 samples (comprising 387 TL and 11 intermediate drug levels) from the induction phase were analyzed; 45 samples (11%) were below the LLOQ. PK profiles were adequately described with a two-compartment model. The estimated inter-patient variability in clearance was 36% and was reduced to 34% when the PK parameters were normalized to body weight (*P* < .01). Strong correlations were observed between the following variables: serum albumin and CRP (*r* = −0.40), CRP and platelet count (*r* = 0.35), CRP and fecal calprotectin (*r* = 0.33), and CRP and white blood cell count (*r* = 0.33). Of these covariates, serum albumin had the largest effect on clearance and was used for subsequent covariate model development. Inclusion of an association between clearance, and both serum albumin and the presence of ADAs improved the model (*P* < .01) and decreased inter-patient variability of clearance to 28%. Inclusion of perianal fistula disease severity significantly improved the model further (ΔOFV = −7.3; *P* = .007); however, inter-patient variability was not reduced. Immunomodulator use was tested as a covariate on clearance and was associated with an 8.7% lower clearance (ΔOFV = −4.0; *P* = .045), but did not meet the pre-specified covariate-inclusion threshold and was therefore not retained in the final model.

Population PK parameter estimates are given in Table 2. For a typical patient with a body weight of 70 kg, serum albumin of 44 g/L and no ADAs, estimated clearance was 0.324 L/day. Clearance increased with body weight (63% from 44 to 125 kg), the presence of ADAs (+319%), and decreasing serum albumin concentrations (+91% from 44 to 22 g/L). Patients with progressive perianal fistulizing disease (TOpClass 2ci, 2cii, and 4) had an estimated 23% increase in infliximab clearance compared to those with no perianal disease or TOpClass 1–2b perianal disease (% relative standard error [%RSE] = 7; Figure 1).

**Figure 1.**
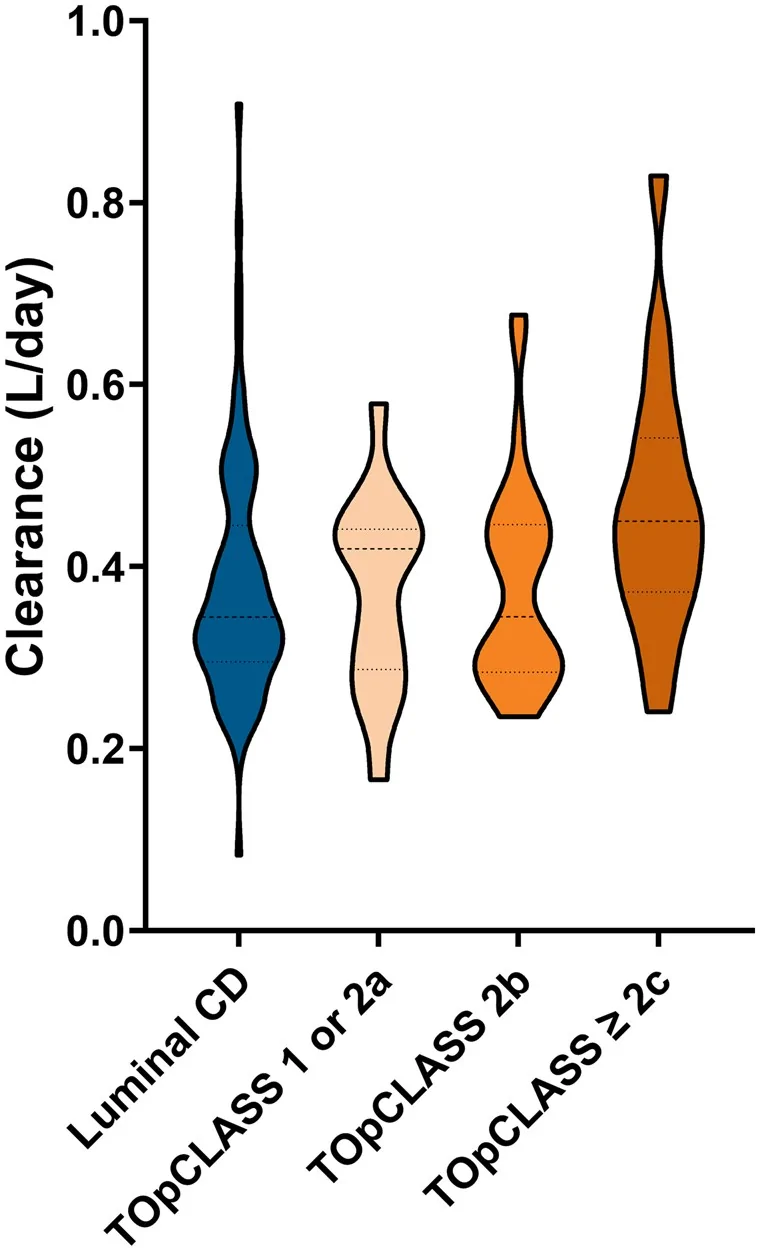
Effect of perianal fistulizing disease on estimated infliximab clearance. Violin plots illustrating the distribution of post-hoc individual clearance estimates of infliximab (empirical Bayes estimates; L/day) stratified by perianal fistulizing disease severity (median, IQR), as defined by TOpClass categories 1 or 2a, 2b, and ≥2c-i. Estimates are based on the final population PK model using induction-phase data. Inclusion of fistula disease activity significantly improved model fit (ΔOFV = −7.3; *P* = .007). No statistical testing was performed on these post-hoc clearance estimates. Note: patients with prior infliximab use and anti-drug antibody formation at week 2 showed clearance >1.0 L/day and were excluded from analysis.

**Table 2.** Final population pharmacokinetic model parameters for infliximab, with precision (%RSE), shrinkage, and bootstrapped estimates.

	Estimate (%RSE) [shrinkage]	Bootstrapped estimate (95% CI)
Parameter		
**CL (L/day)**	0.324 (5)	0.321 (0.293-0.357)
***V*_c_ (L/day)**	4.35 (10)	4.20 (3.45-5.38)
***V*_p_ (L/day)**	1.63 (13)	1.63 (1.24-2.01)
***Q* (L/day)**	0.140 (31)	0.139 (0.088-0.231)
**Covariate effects**		
**Body weight (kg) on CL**	0.465 (32)	0.468 (0.214-0.737)
**Serum albumin (g/L) on CL**	−0.931 (19)	−0.958 (−1.460 to −0.484)
**ADA (presence/absence) on CL**	4.19 (11)	3.84 (2.07-7.76)
**Fistula disease activity on CL** ^a^	1.23 (7)	1.23 (1.02-1.45)
**Body weight (kg) on *V*_c_**	0.385 ^#^	0.385 ^#^
**Body weight (kg) on *V*_p_**	1.08 ^#^	1.08 ^#^
**Body weight (kg) on *Q***	1.26 ^#^	1.26 ^#^
**Inter-individual variability**		
**IIV on CL (%)**	28.0 (7) [6]	27.0 (21.8-32.4)
**Residual error model**		
**Additive error (mg/L)**	0.603 (29)	0.608 (0.046-2.783)
**Proportional error**	0.295 (3)	0.286 (0.132-0.346)

Equations for the main pharmacokinetic parameters; anti-drug antibodies equal 1 if present and 0 if absent, fistula disease activity equals 1 if severe: $CL=0.324\times {\left(\frac{\mathrm{albumin}}{44}\right)}^{-0.931}\times {\left(\frac{\mathrm{weight}}{70}\right)}^{0.465}\times 4.19^{\mathrm{anti}-\mathrm{drug}\;\mathrm{antibodies}}\times 1.23^{\mathrm{fistula}\;\mathrm{disease}\;\mathrm{activity}}$  $V_{c}=4.44\;\times {\left(\frac{\mathrm{weight}}{70}\right)}^{0.385}$, $V_{p}=1.62\;\times {\left(\frac{\mathrm{weight}}{70}\right)}^{1.08}$ and $Q=0.147\;\times {\left(\frac{\mathrm{weight}}{70}\right)}^{1.26}$.

a Fistula disease activity as defined by TOpClass categories: simple or suitable for repair (1 or 2a), symptom control (2b), and progressive disease (2c-, 2c-ii or 4). Abbreviations: CL, clearance; *V*_c_, volume of distribution of the central compartment; *V*_p_, volume of distribution of the peripheral compartment; *Q*, intercompartmental clearance; ADA, anti-drug antibodies; IIV, interindividual variability; %RSE, relative standard error; CI, confidence interval. Notes: parameter estimates are reported with % relative standard error (%RSE) and shrinkage (for IIV parameters). Bootstrap-derived medians and 95% confidence intervals (95% CI) are shown. Covariate effects are expressed as relative changes in clearance. Body weight effects on *V*_c_, *V*_p_, and *Q* were fixed and not re-estimated (#).^24^

The estimated parameters of the final population model were accurate, and the goodness-of-fit plots exhibited no significant deficiencies (Figure S2). The prediction-corrected visual predictive checks indicated that the final model effectively represented both the central tendency and inter-individual variability in drug exposure (Figure S3).

### 3.3. Trough levels and target attainment

Median infliximab TLs were significantly lower in the PFCD group during the induction phase. At week 6, only 33% of PFCD patients achieved target TLs, compared to 51% of LCD patients (*P* = .04). By week 14, median infliximab TLs were 2.9 μg/mL [IQR 1.1-5.5] in PFCD versus 4.8 μg/mL [IQR 1.9-6.6] in LCD (*P* = .04; Figure 2). Target TLs at week 14 were achieved in only 9% of PFCD patients, compared to 46% of LCD patients (*P* < .001), consistent with suboptimal exposure relative to literature-derived thresholds. The proportion of patients reaching predefined target TLs at each time point is shown in Figure 3.

**Figure 2.**
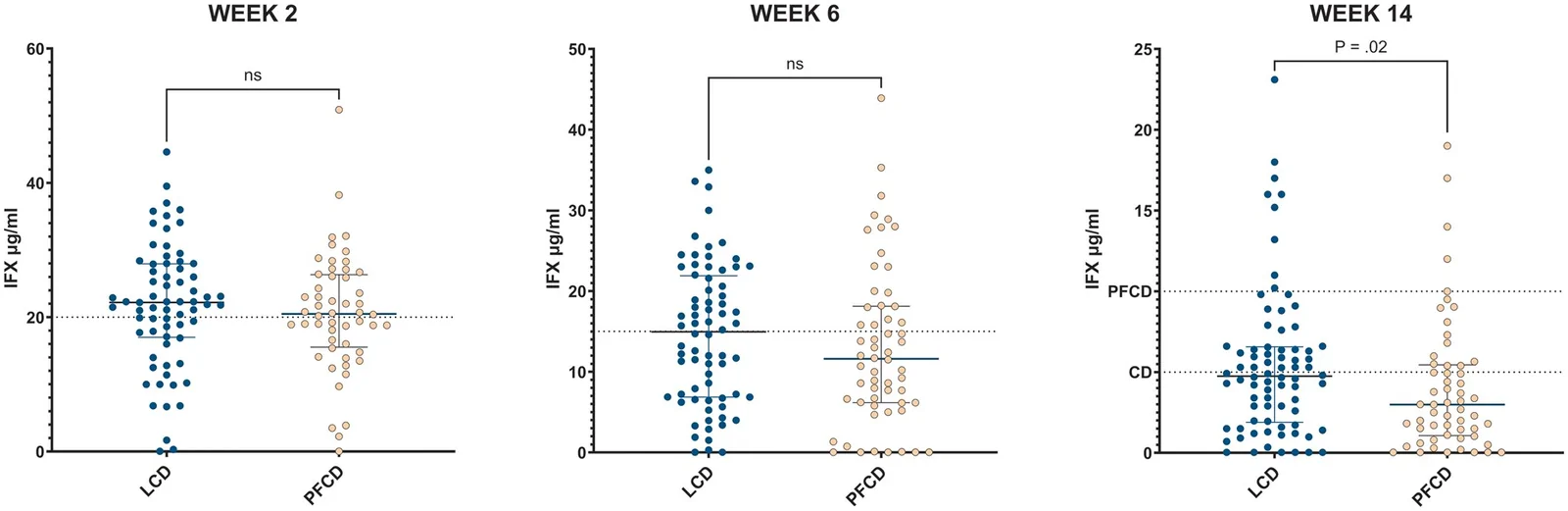
Observed infliximab trough levels at week 2, 6, and 14 in patients with perianal fistulizing Crohn’s disease and luminal Crohn’s disease only. Observed, individual patient IFX trough levels (TL) are plotted for perianal fistulizing Crohn’s disease (PFCD) and luminal Crohn’s disease (LCD) at each timepoint. Horizontal bars indicate median concentrations and interquartile ranges (IQRs). Dashed horizontal lines represent predefined target trough levels for PFCD (20 µg/mL at week 2, 15 µg/mL at week 6, 10 µg/mL at week 14) and CD (5 µg/mL at week 14). Statistical comparisons between PFCD and CD were performed using Mann–Whitney U tests.

**Figure 3.**
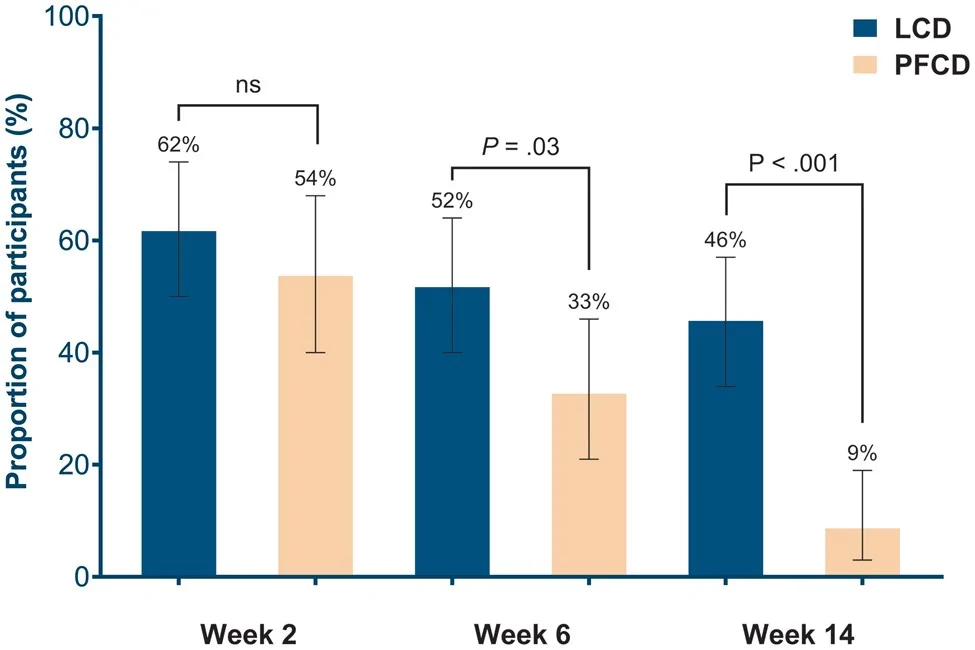
Proportion of patients achieving recommended target trough concentrations. Targets: week 2 for PFCD and LCD = 20 μg/mL, week 6 for PFCD and LCD = 15 μg/mL, week 14 for PFCD = 10 μg/mL and for LCD = 5 µg/mL. Proportions are reported with exact 95% confidence intervals (Clopper–Pearson method), and between-group comparisons were performed using Fisher’s exact test.

In an initial logistic regression model at week 14, PFCD was associated with markedly lower odds of achieving therapeutic infliximab exposure (OR 0.07; 95% CI 0.02-0.22; *P* < .001). This association remained significant in robust Poisson regression with generalized estimating equations (GEEs), which accounts for repeated measures and provides population-averaged risk ratios (aRR 0.14; 95% CI, 0.06-0.45; *P* < .001), independent of induction dose, concomitant immunomodulator use, serum albumin, and fecal calprotectin (Figure S4). Higher induction infliximab doses, body mass index (BMI), and serum albumin levels were independently associated with improved target attainment (Figure S4). To evaluate whether this exposure gap was present throughout induction, we conducted a pooled GEE analysis across weeks 2, 6, and 14 using the same covariate set. PFCD remained independently associated with lower target attainment (aRR, 0.70; 95% CI, 0.52-0.95; *P* = .019), indicating a persistent exposure deficit across the induction phase. These findings remained consistent in sensitivity analyses using uniform therapeutic thresholds (Table S3). As an additional sensitivity analysis, target attainment was numerically similar in both groups (8.8% PFCD vs 11.5% LCD; *P* = .78; Table S4).

Longitudinal infliximab TLs during maintenance therapy were explored up to week 52. Median infliximab TLs remained consistently lower relative to predefined target TLs in PFCD patients compared to those with LCD throughout follow-up, despite changes in dosing and infusion intervals. However, from week 26 onwards, interpretation was limited due to reduced sample sizes (*n* < 20 per group; Figure S5).

### 3.4. Anti-drug antibodies

ADAs against infliximab were detected in 9/18 (50%) PFCD patients and 10/26 (38%) LCD patients with infliximab TL below 1 μg/mL (*P* = .54), with a median time to ADA development of 20 weeks [IQR 9-28]. Median ADA levels were comparable between PFCD (250 AU/mL, IQR 92-565) and LCD patients (157 AU/mL, IQR 74-454; *P* = .47). At week 14, 22% of PFCD patients had very low infliximab TLs <1 μg/mL, compared to 10% in LCD patients (*P* = .07).

Among ADA-positive patients, 35% exhibited a transient immunogenic response, which may have been influenced by subsequent drug intensification. Three patients showed unusually high clearance values (>1.0 L/day), which were associated with the presence of high-titer ADAs at week 2; all of these patients had previously used infliximab.

## 4. Discussion

In this large prospective cohort evaluating intravenous infliximab PK in patients with PFCD, we observed substantial early infliximab underexposure despite standard induction dosing: only 9% of PFCD patients achieved therapeutic TLs at week 14, compared to 46% of patients with LCD. Population PK modeling identified a 23% increase in infliximab clearance in patients with progressive perianal fistulizing disease, independent of albumin, body weight, and ADA formation. These findings provide mechanistic support for the observed underexposure and reinforce the rationale for intensified induction strategies in PFCD.

Enhanced clearance in PFCD likely reflects overlapping mechanisms, including systemic inflammation, high TNF-α burden, and local tissue factors that may increase antibody catabolism or local sequestration.^20^^,^^25^^,^^38^ Proteolytic inactivation of infliximab has been demonstrated in inflamed IBD mucosa, raising the possibility of similar processes in perianal tissue.^39^ Although our PK model did not support an expanded distribution volume as a major contributor, theoretical mechanisms such as impaired neonatal Fc receptor recycling, immune-mediated clearance, or protein loss via draining fistulas may also contribute.^40–42^

Kinetic profiling showed no difference in TLs at week 2, with disparities emerging by week 6 and becoming significant by week 14, indicating similar initial distribution, but accelerated elimination in PFCD over time. These findings are consistent with prior studies linking low post-induction TLs to worse clinical outcomes, increased immunogenicity, and failure to achieve deep remission in CD, including PFCD.^23^^,^^25^^,^^28^^,^^29^ Furthermore, only a 10 mg/kg 4-weekly intensified dose was able to achieve therapeutic target TLs in >50% of patients who had subtherapeutic post-induction TLs in a PK simulation model.^43^ Although clearance differences across the entire PFCD cohort did not reach statistical significance, the trend toward higher clearance likely reflects a modest true effect, consistent with low TLs and target attainment throughout the cohort. This robustness was threshold-dependent: the PFCD–LCD difference persisted at a uniform 5 µg/mL threshold but attenuated at 10 µg/mL, a target not established for LCD, supporting disease-appropriate thresholds or continuous trough concentrations over a single uniform cutoff. Despite a low overall incidence of ADAs, persistently lower TLs may predispose PFCD patients to future immunogenicity and secondary loss of response.

In the absence of robust medical alternatives for managing PFCD, biologically plausible and safe interventions such as intensified induction dosing warrant consideration. Intensified infliximab regimens have not been associated with increased adverse events in IBD cohorts,^44^^,^^45^ and front-loaded induction strategies (7.5-10 mg/kg at weeks 0, 2, and 6) combined with early proactive TDM may improve target attainment, short-term efficacy, and long-term durability. This approach is supported by superior 12-month outcomes with a front-loaded 10 mg/kg induction regimen in PFCD compared to standard dosing, including higher clinical fistula healing rates (82% vs 45%), fewer reinterventions (12% vs 42%), and improved achievement of target maintenance TLs (>10 µg/mL in 82% vs 5%).^32^ Early TDM allows timely identification of underexposure and dose optimization, although available pharmacodynamic evidence remains observational and cannot confirm a causal relationship.^46^ This is a broader challenge in the TDM literature, where treatment intensification often follows PK results, creating inherent confounding.^47^ TOpClass severity may guide clinicians as an accessible proxy for anticipated PK burden, helping to identify which patients with PFCD are most likely to require intensified induction dosing pre-emptively, before subtherapeutic exposure occurs.

So far, no interventional trials have directly compared intensified versus standard infliximab induction in PFCD. In NOR-DRUM, proactive TDM improved outcomes during maintenance (NOR-DRUM B) but not during induction (NOR-DRUM A) treatment among patients with immune-mediated inflammatory diseases.^48^^,^^49^ However, only a minority of patients in NOR-DRUM A had protocol-guided induction dose adjustments, while in PFCD we observe >90% subtherapeutic levels by week 14. This supports a pragmatic strategy of front-loaded induction with early TDM, and also highlights the need for PFCD-specific randomized trials. Future studies should address three priorities: (1) randomized trials directly comparing intensified versus standard induction in PFCD, (2) tissue-level PK and distribution studies to quantify local exposure and drug loss, and (3) evaluation of TL thresholds that predict surgical success and long-term remission, which may be essential or even a prerequisite for definitive surgical therapy. This is an important avenue for translational research, particularly as multidisciplinary approaches to PFCD evolve. The ongoing PROACTIVE trial will be pivotal in evaluating whether individualized infliximab dosing based on proactive TDM improves for outcomes including fistula healing, recurrence-free survival, and quality of life.^30^

The strengths of this study include its prospective design, validated population PK modeling, and a well-matched control group of LCD patients. Perianal fistulizing disease severity was classified using TOpCLass categories, allowing standardized and reproducible stratification of disease severity across clinically meaningful phenotypes, including aggressive and cutaneous forms. This enhances interpretability and comparability with existing literature. The large cohort for a population PK analysis, comprising 148 patients with extensive longitudinal sampling, was based on simulation-based recommendations from prior population PK studies in IBD and allowed robust estimation of infliximab clearance and covariate effects with acceptable precision. Our findings provide actionable insights for clinical practice, which could potentially improve outcomes in this difficult to treat patient population with limited effective medical options. Furthermore, by establishing a disease-specific PK profile, this study contributes to the fundamental understanding of infliximab disposition in complex Crohn’s phenotypes, offering new directions for translational and mechanistic research.

Limitations include the single-center design, conducted in a tertiary IBD center with a dedicated fistula clinic. However, our cohort included a wide range of PFCD phenotypes, from mild to highly complex disease, increasing applicability. Although care paths were standardized, immunomodulator use was not uniform between groups. Immunomodulator use was tested as a covariate on clearance but did not meet our pre-specified inclusion threshold and was therefore not retained in the final model. Perianal fistula disease activity remained significantly associated with clearance independent of this and other tested covariates. This highlights that immunomodulator use alone is unlikely to explain the increased infliximab clearance in PFCD. Furthermore, the large majority of PFCD patients in our cohort presented with complex fistulizing disease (50/62, 81%, by AGA classification),^36^ precluding a statistically meaningful comparison between simple and complex disease subgroups within this study.

In conclusion, severe PFCD is characterized by increased infliximab clearance leading to early underexposure during induction. These PK findings provide a mechanistic rationale for empirically applying an intensified induction regimen in this high-risk population, complementing existing pharmacodynamic evidence. Future research should aim to refine these strategies through mechanistic tissue studies and evaluate long-term clinical and surgical outcomes explicitly linked to pharmacokinetically informed dosing regimens.

## Supplementary Material

jjag151_Supplementary_Data

## Data Availability

All data relevant to the study are included in the article or uploaded as supplementary information. Individual participant data are not publicly available due to privacy and ethical restrictions, as these data cannot be fully de-identified. Further information may be available upon reasonable request and subject to review by the appropriate ethics committee and data protection regulations.

## Conference presentation

This work was presented at European Crohn’s and Colitis Organization Congress 2025 and United European Gastroenterology Week 2025.
