# Supplementary material for: Perianal fistulizing Crohn’s disease increases infliximab clearance and causes low infliximab exposure (ATLANTIC)
Source: J Crohns Colitis. 2026 Oct 23;20(10):jjag151. doi: 10.1093/ecco-jcc/jjag151 (PMC13692796; doi:10.1093/ecco-jcc/jjag151)
Supplement: Supplementary file 1 [file jjag151_supplementary_data.zip › Supplementals_JCC_25226_rev_final_clean.docx]

**Supplementary materials**

**Perianal Fistulizing Crohn’s Disease Increases Infliximab Clearance and Leads to Lower Serum Concentrations Compared to Luminal Crohn’s Disease**

- Supplementary table S1. Predefined covariate-parameter relationships
- Supplementary table S2. Available pre-infusion serum samples across the timepoints and cohorts
- Supplementary Table S3. Adjusted risk ratios for achieving therapeutic infliximab TLs during induction, using uniform and phenotype-specific targets
- Supplementary Table S4. Sensitivity analysis: infliximab target attainment at week 14 using a uniform 10 µg/mL threshold in both PFCD and LCD
- Supplementary figure S1. Study enrollment and analysis flowchart
- Supplementary figure S2. Goodness-of-fit plots for the PK model
- Supplementary figure S3. Prediction-corrected visual predictive check plots for the PK model
- Supplementary figure S4. Adjusted risk ratios for achieving infliximab targets at week 14
- Supplementary figure S5. Longitudinal observed IFX TL trends during maintenance phase (week 14 to 50)

Supplementary table S1. Predefined covariate-parameter relationships

| Covariate | Time-dependency | Parameter | Rationale |
| --- | --- | --- | --- |
| Body weight | Time-dependent | CL, V1, V2, Q | Weight-based dosing |
| Albumin | Time-dependent | CL | Marker of disease activity |
| C-reactive protein | Time-dependent | CL | Marker of disease activity |
| Fecal calprotectin | Time-dependent | CL | Marker of disease activity |
| White blood cell count | Time-dependent | CL | Marker of disease activity |
| Platelet count | Time-dependent | CL | Marker of disease activity |
| Anti-drug antibodies, presence | Time-dependent | CL | Prior knowledge |
| Smoking | Time-independent | CL | Prior knowledge |
| Sex | Time-independent | CL | Prior knowledge |
| Perianal fistula disease severity | Time-independent | CL | Clinical interest |

Supplementary table S2. Available pre-infusion serum samples across the timepoints and cohorts

|  | W2 | W6 | W14 | after W14 |
| --- | --- | --- | --- | --- |
| PFCD, n (% N)  (N = 62) | 51 (82) | 57 (92) | 57 (92) | 160 |
| LCD, n (% N)  (N = 86) | 70 (81) | 72 (84) | 80 (93) | 209 |

Supplementary Table S3. Adjusted risk ratios for achieving therapeutic infliximab TLs during induction, using uniform and phenotype-specific targets

| Targets | Covariate | aRR | 95% CI | P-value |
| --- | --- | --- | --- | --- |
| Week 14 uniform target TLs | **PFCD vs LCD** | **0.58** | **0.36 – 0.95** | **.029** |
|  | IFX dose (mg/kg) (per SD) | 1.16 | 0.96 – 1.40 | .12 |
|  | Immunomodulator use (yes/no) | 1.00 | 0.99 – 1.00 | .39 |
|  | Fecal calprotectin (µg/g) (per SD) | 1.16 | 0.96 – 1.41 | .13 |
|  | Serum albumin (g/L) (per SD) | 1.14 | 0.90 – 1.44 | .29 |
| Overall induction uniform target TLs | **PFCD vs LCD** | **0.70** | **0.52 – 0.95** | **.019** |
|  | **IFX dose (mg/kg) (per SD)** | **1.14** | **1.01 – 1.28** | **.030** |
|  | Immunomodulator use (yes/no) | 1.02 | 0.996 – 1.05 | .10 |
|  | Fecal calprotectin (µg/g) (per SD) | 1.03 | 0.91 – 1.18 | .61 |
|  | **Serum albumin (g/L) (per SD)** | **1.21** | **1.02 – 1.43** | **.030** |
| Overall induction phenotype target TLs | **PFCD vs LCD** | **0.59** | **0.44 – 0.80** | **< .001** |
|  | **IFX dose (mg/kg) (per SD)** | **1.13** | **1.01 – 1.26** | **.03** |
|  | Immunomodulator use (yes/no) | 1.03 | 1.00 – 1.05 | .10 |
|  | Fecal calprotectin (µg/g) (per SD) | 1.03 | 0.90 – 1.17 | .69 |
|  | **Serum albumin (g/L) (per SD)** | **1.20** | **1.00 – 1.42** | **.04** |

Adjusted risk ratios (aRRs), 95% confidence intervals (CIs), and P-values from robust Poisson regression models with generalized estimating equations (GEE) comparing patients with perianal fistulizing Crohn’s disease (PFCD) to those with luminal Crohn’s disease (LCD). Response was defined as achieving predefined infliximab (IFX) trough level thresholds at week 14 or across the full induction period (weeks 2, 6, and 14). Three models are shown: (1) Week 14 analysis using a uniform week-14 therapeutic threshold (TL ≥5 µg/mL), (2) pooled analysis across induction using uniform thresholds (week 2: 20 µg/mL, week 6: 15 µg/m, week 14: 5 µg/mL), and (3) pooled analysis using phenotype-specific thresholds (week 14: TL ≥10 µg/mL for PFCD, ≥5 µg/mL for LCD). All models adjust for scaled IFX dose (mg/kg), immunomodulator use, serum albumin, and fecal calprotectin. Significant associations are highlighted in bold.

Supplementary Table S4. Sensitivity analysis: infliximab target attainment at week 14 using a uniform 10 µg/mL threshold in both PFCD and LCD

|  | PFCD | LCD | P-value |
| --- | --- | --- | --- |
| Target attainment at week 14, n/N (%) [95% CI] | 5/57 (8.8) [2.9–19.3] | 9/78 (11.5) [5.4–20.8] | .78 |

*Fisher's exact test. 95% confidence intervals calculated using the Clopper-Pearson method, consistent with the primary analysis (Figure 3). This sensitivity analysis was performed to assess the robustness of the primary target-attainment findings.

Supplementary figure S1. Study enrollment and analysis flowchart

A total of 926 patients from the Amsterdam UMC IBD Biobank (Future IBD cohort) were screened. 148 patients with a confirmed diagnosis of CD started intravenous infliximab and were eligible for the intention-to-treat (ITT) analysis and stratified by disease phenotype: 62 patients with perianal fistulizing Crohn’s disease (PFCD) and 86 patients with luminal Crohn’s disease (LCD) only.

Supplementary figure S2. Goodness-of-fit plots for the PK model

Goodness-of-fit plots for the final population pharmacokinetic model of infliximab. Observed concentrations are plotted against population and individual predictions (top panels). Weighted residuals (IWRES and CWRES) are plotted against individual predictions and time after dose (bottom panels). The model adequately describes the observed data without evidence of systematic bias

Supplementary figure S3. Prediction-corrected visual predictive check plots for the PK model

Visual predictive checks (VPC) for the final population pharmacokinetic model of infliximab. The observed data (blue circles) are overlaid with the model-predicted median (solid red line) and 90% prediction intervals (shaded red and blue areas). The dashed red lines represent the 5^th^ and 95^th^ percentiles of the observed data. The model adequately described the time course and variability of infliximab concentrations during the induction phase.

Supplementary figure S4. Adjusted risk ratios for achieving infliximab targets at week 14

Multivariable Poisson generalized estimating equations (GEE) analysis showing adjusted risk ratios (aRRs) with 95% confidence intervals (CIs) for infliximab induction dose (per standard deviation increase), immunomodulator use (IMM), serum albumin concentration (per standard deviation increase), fecal calprotectin concentration (per standard deviation increase), and the interaction between PFCD and week 14. Risk ratios are plotted on a logarithmic scale. Risk ratios <1 indicate a lower probability of achieving target trough levels.

Supplementary figure S5. Longitudinal observed IFX TL trends during maintenance phase (week 14 to 50)

Longitudinal median infliximab trough levels (µg/ml) from week 14 to week 50 in patients with PFCD and luminal CD only. Dotted lines indicate standard target trough levels (≥ 5 µg/ml for CD, ≥ 10 µg/ml for PFCD). Data beyond week 26 are based on smaller sample sizes (n < 20 per group) and should be interpreted with caution.

Supplementary results.

Among PFCD patients with fistula status assessed at week 14, individual post-hoc clearance estimates were 8% higher in patients with active fistulizing disease compared with those in clinical response or remission (ΔOFV = -2.4; P=.12; 95% CI ±10%). Observed infliximab TLs at week 14 were lower in patients with active disease (median 1.8 µg/mL, IQR 1.0–4.4, n=17) than in those with clinical response or remission (median 6.0 µg/mL, IQR 3.6–9.6, n=24; P=.007), with a graded pattern across individual status categories (active disease 1.8 µg/mL; clinical response 5.0 µg/mL; clinical remission 6.6 µg/mL). These exploratory, cross-sectional analyses should be interpreted with caution: fistula status was not assessed on a fixed, prospectively defined schedule.
